# Chemical and Mechanical Stability of Rotary-Die-Extruded Recycled PET Fibers Under Aggressive Aqueous Environments

**DOI:** 10.3390/polym18161980

**Published:** 2026-08-14

**Authors:** Rabeh Slimani, Sahnoun Zengah, Ismail Drai, Abdelghani Baltach, Rachid Sahnoun, Habib Merouane, Dursun Murat Sekban, Ecren Uzun Yaylacı, Orkun Burak Öztürk, Murat Yaylacı

**Affiliations:** 1 Department of Mechanical Engineering, University of Mustapha Stambouli, Mascara 29000, Algeria; slimanirabeh1@gmail.com; 2 Laboratory of Mechanics, Structure & Energy (LMSE), University of Mouloud Maameri, Tizi Ouzou 15000, Algeria; s.zengah@univ-mascara.dz (S.Z.); ismail.drai@gmail.com (I.D.); r_sahnoun@yahoo.fr (R.S.); merouanehabib@yahoo.fr (H.M.); 3 Mechanics of Materials, Energy and Environment Laboratory (L2M2E), University of Mustapha Stambouli, Mascara 29000, Algeria; abdelghani.baltach@univ-tiaret.dz; 4 Department of Mechanical Engineering, University of Ibn Khaldoun, Tiaret 14000, Algeria; 5 Department of Marine Engineering Operations, Karadeniz Technical University, 61080 Trabzon, Türkiye; msekban@ktu.edu.tr; 6 Trabzon Teknokent, WMS Engineering Services Industry Trade Limited Company, 61080 Trabzon, Türkiye; 7 Faculty of Fisheries, Recep Tayyip Erdogan University, 53100 Rize, Türkiye; ecren.uzunyaylaci@erdogan.edu.tr; 8 Turgut Kıran Maritime Faculty, Recep Tayyip Erdogan University, 53900 Rize, Türkiye; orkunburak.ozturk@erdogan.edu.tr; 9 Department of Civil Engineering, Recep Tayyip Erdogan University, 53100 Rize, Türkiye

**Keywords:** recycled polyethylene terephthalate, rotary die extrusion, chemical stability, mass retention, tensile strength, aqueous exposure, textile recycling

## Abstract

This study evaluated the chemical and mechanical stability of recycled polyethylene terephthalate (rPET) fibers produced by rotary die extrusion under selected aqueous exposure conditions. The rPET fibers, natural wool, cotton batting, and a polyester and cotton apparel fabric were exposed to powder detergent, liquid soap, bleach, and dilute hydrochloric acid for periods ranging from 2 h to 7 days. Mass retention was assessed for all materials, whereas tensile properties were evaluated only for rPET. The rPET fibers retained between 99.92 and 100% of their initial mass and more than 95% of their initial tensile strength after 7 days of exposure. Wool showed its greatest mass loss in bleach, while cotton batting and apparel fabric showed measurable mass losses under specific exposure conditions. These reference materials differed in composition and physical form and were therefore used only to provide descriptive context. Overall, the results indicate high mass retention and tensile property retention of the investigated rPET fibers under static exposure at 25 °C. The findings support their potential use in textile applications requiring resistance to the tested aqueous environments but do not establish molecular chain integrity or equivalence with virgin PET.

## 1. Introduction

The textile industry, one of the most significant and dynamic sectors globally, faces growing environmental challenges related to overconsumption, waste generation, and the intensive use of natural resources [1,2,3,4,5]. Global production of textile fibers has increased considerably in recent decades, with synthetic fibers accounting for a predominant share [6,7,8,9]. In this context, textile recycling and the use of recycled fibers have become imperatives to promote a circular economy and reduce the industry’s ecological footprint [10,11,12,13,14]. Among synthetic polymers, polyethylene terephthalate (PET) is widely used, particularly for packaging, and its conversion into recycled PET (rPET) fibers offers a promising route for valorizing plastic waste [15,16,17,18,19,20]. Established mechanical recycling routes include bottle collection, sorting, washing, shredding into flakes or chips, remelting, pellet production, and conversion into films or fibers through extrusion and melt spinning. Beyond mechanical recycling, PET waste can also be processed through chemical recycling routes that recover monomers or oligomers for subsequent polymer production. Jakić et al. reported that extrusion-based reprocessing of post-consumer PET caused no significant change in its chemical structure or thermal stability, although changes in melting and crystallization behavior were observed. This finding indicates that processing history can influence the thermal characteristics of recycled PET [21].

Recycled PET fibers are increasingly employed in various applications, ranging from apparel to technical textiles, due to their interesting mechanical properties and potential durability [22,23,24]. However, to ensure their performance and longevity, especially in applications subjected to rigorous usage and maintenance conditions, it is crucial to evaluate their behavior when exposed to various chemical agents. Textiles are frequently exposed to detergent solutions during washing cycles, and their resistance to these products is a key factor in their durability [25,26,27,28]. Low chemical resistance can lead to premature fiber degradation, mass loss, alteration of mechanical properties, and a reduction in the textile product’s lifespan. This aspect is particularly critical for engineered fibers designed for repeated or intensive cleaning cycles, where dimensional stability and integrity must be maintained over time. PET contains ester bonds that can undergo hydrolysis under sufficiently severe conditions. Strong acidic or alkaline media may promote chain cleavage, while alkaline treatment can also produce surface deweighting through progressive ester hydrolysis. Enzymatic degradation follows a related mechanism through ester bond cleavage at accessible polymer surfaces. The extent of these processes depends on pH, temperature, exposure time, water accessibility, and polymer morphology.

Within these recycling routes, rotary die extrusion may influence rPET fiber diameter, morphology, microstructure, surface characteristics, and chemical response [29]. Although several studies address the mechanical or thermal behavior of rPET, direct evaluations of fibers produced by a defined rotary die extrusion route under identical aqueous exposure conditions remain limited. Most available studies also do not connect processing history with both mass retention and tensile property retention after exposure to detergents, oxidizing agents, or acidic solutions. Natural and blended textile materials may provide practical context for assessing the serviceability of rPET in textile applications [30,31,32,33], but their different compositions and physical forms prevent them from serving as fully equivalent controls for rPET. Accordingly, the primary scientific focus of the present work is the stability of rotary-die-extruded rPET fibers.

This study therefore focuses on the chemical and mechanical stability of rPET fibers produced by rotary die extrusion after exposure to powder detergent, liquid soap, bleach, and dilute hydrochloric acid. Mass retention is assessed for rPET and three reference textile materials, whereas tensile properties are evaluated only for rPET. The reference materials are included solely to provide a descriptive practical context and not to establish direct mechanistic equivalence. This study aims to determine whether the tested rPET fibers retain mass and tensile properties under the selected aqueous conditions. Virgin PET was not included as an experimental control, and no direct equivalence between recycled and virgin PET is claimed. Overall, this work provides a systematic dataset on rPET stability. The conclusions are restricted to the evidence provided by the mass and tensile measurements conducted in this study.

## 2. Experimental Procedures

This study evaluated the chemical stability of recycled PET fibers produced by rotary die extrusion under four aqueous exposure conditions. Mass retention was determined after exposure to powder detergent, liquid soap, bleach, and hydrochloric acid for 2 h, 24 h, 3 days, and 7 days. Natural wool, cotton batting, and apparel fabric were included only as descriptive reference materials. Because these materials differ in composition and physical form, they were not treated as equivalent mechanistic controls for rPET. Mechanical evaluation was limited to the rPET fibers.

**Figure 1:**
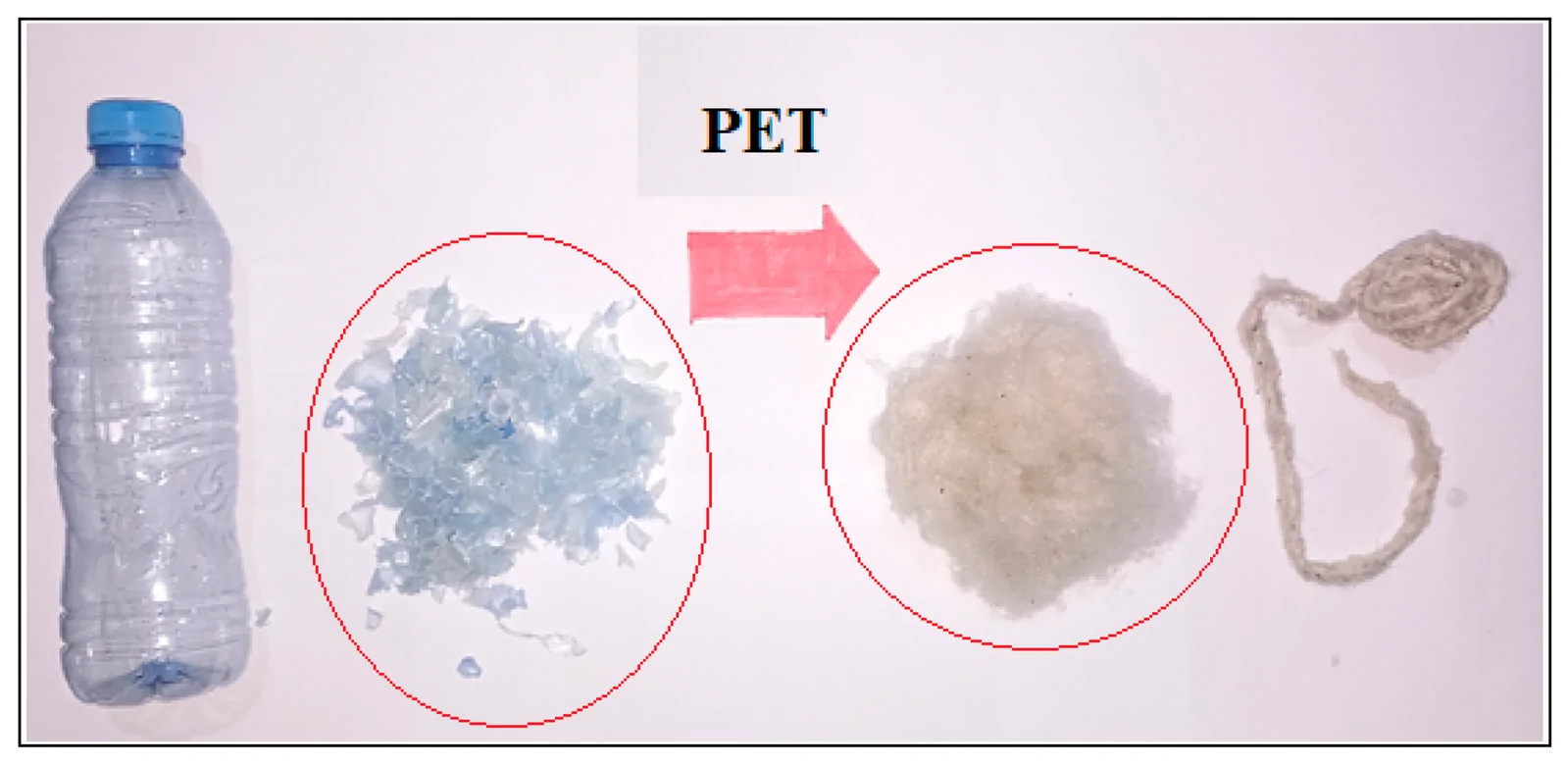
Overview of PET bottle recovery and conversion from flakes to fibers and yarn.

The production of the rPET fibers began with the collection of post-consumer PET bottle waste. The bottles were transported, sorted by color and density, washed to remove contaminants, shredded into flakes, sorted according to quality, and dried. The cleaned flakes were subsequently melted and converted into rPET pellets. For this study, the pellets were processed using a rotary die extrusion machine operated at a barrel temperature of 260 °C, a screw rotation speed of 45 rpm, and a die opening diameter of 1 mm. The extruded filaments were cooled under ambient conditions and cut into staple fibers with lengths of approximately 10 to 12 mm. Figure 1 presents an overview of the PET bottle recycling and fiber production process.

PET is a thermoplastic polyester with the repeating chemical formula (C_10_H_8_O_4_)*_n_*. Typical physical and chemical properties of PET are summarized in Table 1. These values describe the general characteristics of PET and do not constitute direct structural characterization of the rPET fibers produced in this study.

### 2.1. Materials and Products Used

The experimental protocol relied on specific equipment to conduct the evaluations. A precision electronic balance was essential for accurately quantifying the mass variations in the samples after exposure to the different solutions. The balance used had a resolution of 0.001 g, allowing precise detection of small mass losses. Individual plastic beakers were used for each sample, allowing for controlled immersion and preventing cross-contamination. Each test specimen had an initial dry mass of 10.000 g. The materials included natural wool, cotton batting, an apparel fabric, and the rPET staple fibers produced by rotary die extrusion. The rPET fibers constituted the primary material investigated in this study.

The cotton batting consisted of medical-grade absorbent cotton composed of 100% purified cellulose and was obtained from a local paramedical equipment supplier. Natural wool fibers were obtained from local sheep breeds at a local agricultural farm in the Mascara region, Algeria, during routine seasonal shearing, with permission from the farm owners. The wool was collected as a byproduct of routine farming practice, and no animals were handled by the researchers or subjected to any experimental procedure for this study. The apparel fabric was purchased from a local commercial textile supplier and consisted of a standard industrial polyester/cotton blend composed of 65% polyester and 35% cotton, with a twill structure and an areal density of approximately 180 g/m^2^. The recycled PET fibers were produced in-house from post-consumer PET water bottles collected from local waste streams, which were cleaned, shredded into flakes, and processed using the rotary die extrusion system.

All samples were conditioned at 21 ± 2 °C and 65 ± 5% relative humidity for 24 h to reduce moisture-related variation in the mass measurements. Before testing, the wool fibers were scoured using a nonionic detergent and rinsed several times with distilled water to remove lanolin and organic impurities. The cotton batting and apparel fabric were not subjected to a separate scouring treatment. All materials, including the rPET fibers, were rinsed with distilled water and oven dried at 60 °C until constant mass. Test portions with an initial dry mass of 10.000 g were then prepared.

Apparel fabrics were cut into 5 × 5 cm swatches to ensure uniform surface-area-to-volume ratios, whereas loose fibers (cotton, wool, rPET) were tested in staple form. The different physical forms and surface area to volume ratios may influence reagent diffusion. Therefore, the results obtained for the reference materials were used only for descriptive comparison and not as evidence of directly equivalent degradation kinetics.

Four aqueous media were selected to represent surfactant containing cleaning conditions, oxidative exposure, and strongly acidic exposure. Commercial formulations were used according to the concentrations listed in Table 2. Their trade names and available label-based specifications are reported to improve reproducibility. No independent chemical composition analysis of the commercial products was performed.

Powder detergent and liquid soap were selected to represent concentrated cleaning media. Bleach was selected as an oxidative challenge medium, while hydrochloric acid represented severe acidic exposure. The concentration of 100 g/L used for the detergent and liquid soap was intentionally higher than normal laundering concentrations. It was selected as an accelerated screening condition to increase the likelihood of detecting mass changes during the seven-day exposure period. Therefore, these conditions should not be interpreted as a direct simulation of domestic laundering.

The four solutions were prepared according to the concentrations specified in Table 2. Each sample was weighed to an initial mass of 10.000 g and individually immersed in 50 mL of the corresponding solution in a separate beaker. The solution to sample ratio was therefore 5 mL/g. This ratio provided an excess volume of solution relative to the sample mass and reduced the likelihood of substantial concentration changes caused by reagent consumption.

Exposure to the solutions was conducted for progressive durations of 2 h, 24 h, 3 days, and 7 days. All immersion experiments were performed at controlled room temperature (25 ± 1 °C) without agitation, ensuring identical diffusion-based exposure conditions across all samples. At the end of each exposure period, the samples were carefully recovered, rinsed abundantly with clear water to remove any solution residue, and then oven-dried at 60 °C until constant mass. Constant mass was defined as a mass variation below 0.001 g between two consecutive measurements.

**Figure 2:**
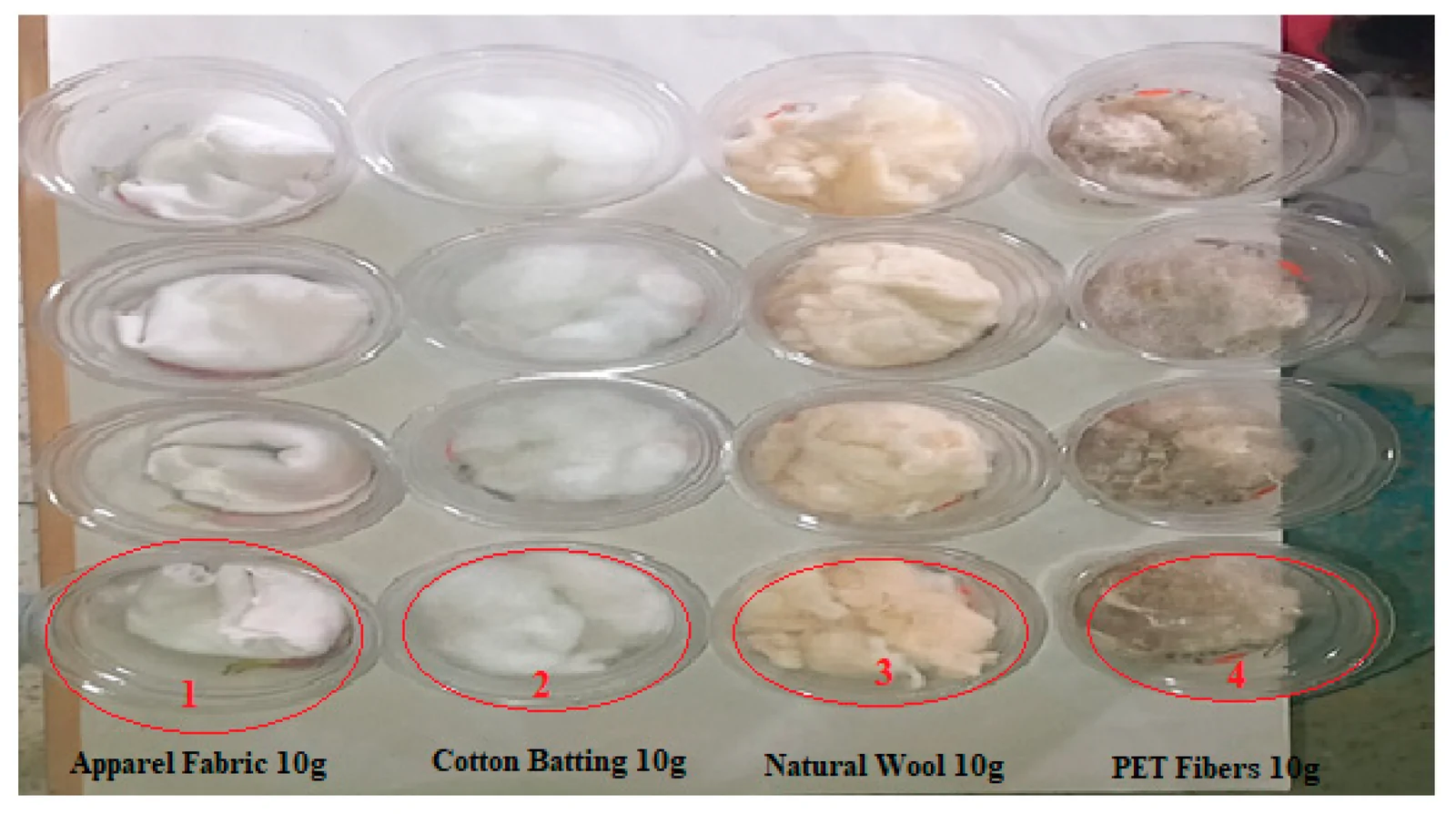
The four types of textile fibers studied before immersion.

A final weighing was then performed to accurately assess the mass loss sustained by each fiber. The percentage mass loss was calculated as follows:

$$Mass\;Loss\;\left(\%\right)=\frac{m_{0}-m_{f}}{m_{0}}\;\times \;100$$

where m_0_ is the initial dry mass and m_f_ is the final dry mass after exposure, rinsing, and drying. All measurements were conducted in triplicate for each test condition, and the results were reported as mean values with standard deviations. The visual appearance of the four textile fiber types prior to chemical immersion is presented in Figure 2.

### 2.2. Mechanical Evaluation of rPET Fibers

Mechanical characterization was conducted only for untreated rPET fibers and rPET fibers after 7 days of exposure to each solution. Before mechanical testing, the exposed fibers were rinsed with distilled water and dried to constant mass under the same conditions used for the mass retention measurements. Tensile strength, Young’s modulus, and elongation at break were determined after exposure, rinsing, and drying. The untreated rPET fibers were used as the reference condition for evaluating changes caused by chemical exposure. Strength retention was calculated relative to the tensile strength of the untreated rPET fibers using the ratio of the tensile strength after exposure to the initial tensile strength, expressed as a percentage. The resulting values were used to assess whether chemical exposure caused measurable changes in load bearing capacity, stiffness, or deformation at break.

In parallel, the surface appearance of the untreated and exposed rPET fibers was examined by optical microscopy. The fibers exposed for 7 days were qualitatively evaluated for visible fibrillation, pitting, cracking, surface roughening, and loss of transparency. This observation was used only as supporting qualitative evidence because optical microscopy cannot detect subtle structural or molecular changes.

These measurements were used to evaluate the retention of mechanical properties within the rPET group and were not used for direct mechanical comparison with wool, cotton batting, or apparel fabric. Accordingly, the mechanical results were interpreted only in relation to the untreated rPET control and the different chemical exposure conditions.

## 3. Results and Discussion

### 3.1. Microscopic Observations of PET Fibers

**Figure 3:**
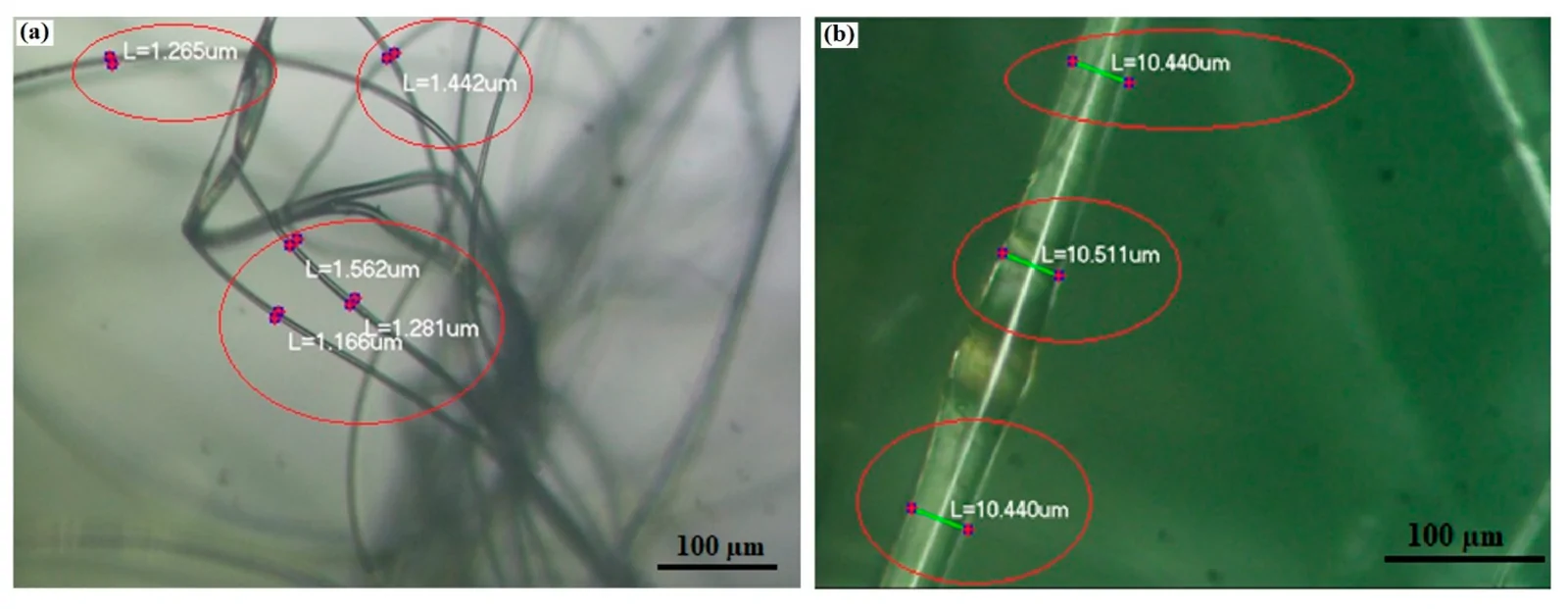
(**a**) Fine rPET fibrils and (**b**) a larger rPET fiber with longitudinal striations.

Representative microscopic images of recycled PET fibers with different diameters and surface morphologies are shown in Figure 3. The images indicate variability in the apparent diameter and surface appearance of the produced rPET fibers. Figure 3a highlights an entanglement of fine PET fibrils with measured diameters ranging from approximately 1.1 to 1.6 µm. These fine and translucent fibrils contribute to the heterogeneous morphology observed within the sample. Figure 3b presents an individual PET fiber with a larger diameter of approximately 10.4 to 10.5 µm. This fiber is characterized by pronounced longitudinal striations. These striations may represent surface features generated during extrusion and cooling. However, optical microscopy alone cannot confirm molecular orientation or crystallinity. The surface of this fiber, apart from the striations, appears relatively smooth, with good overall diameter uniformity despite a slight localized swelling.

### 3.2. Mass Loss Analysis and General Trends

**Figure 4:**
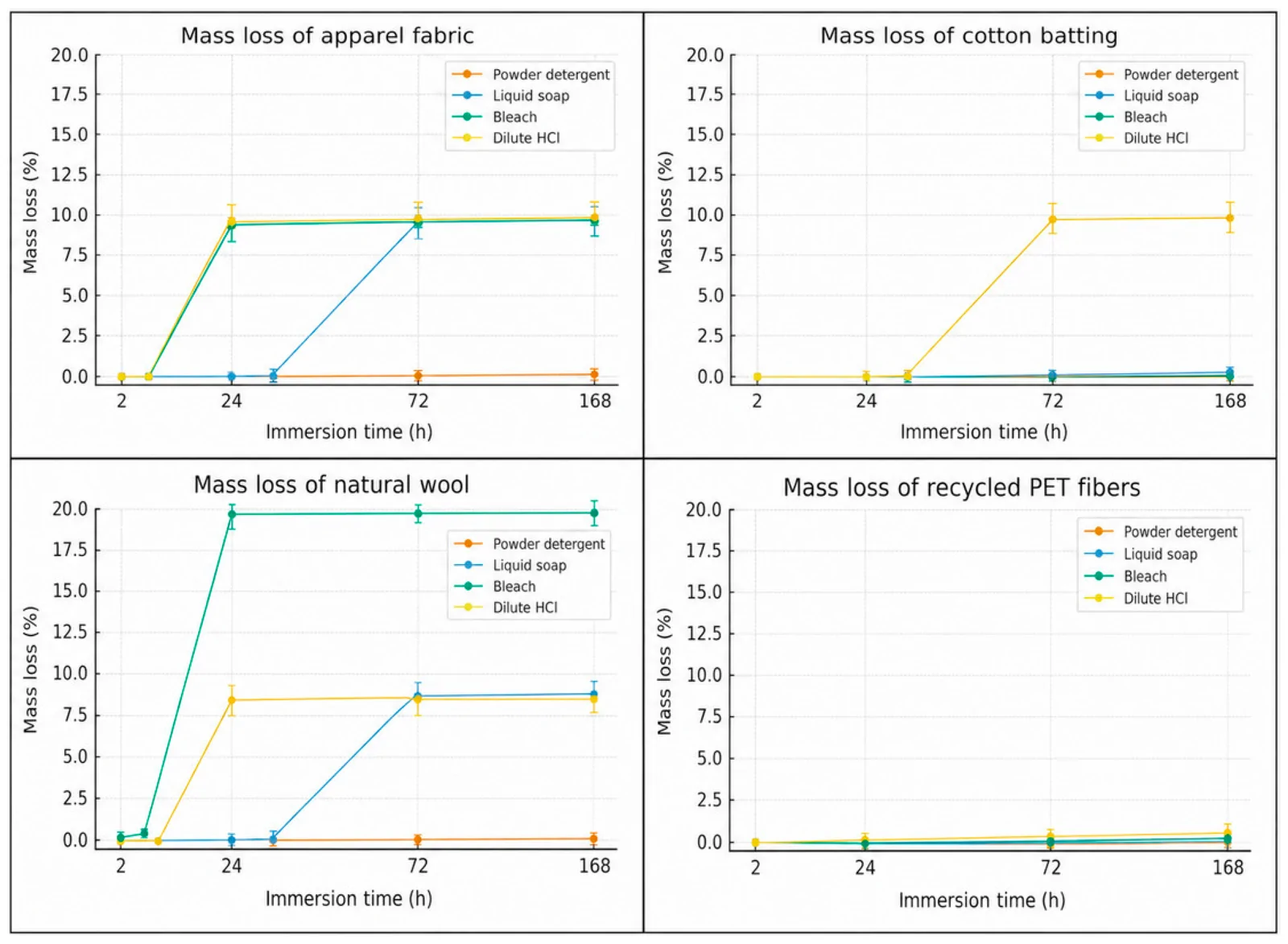
Mean mass loss of apparel fabric, cotton batting, natural wool, and rPET fibers. Error bars represent standard deviation, *n* = 3.

The mass-loss behavior of all textile samples was evaluated after immersion in four different chemical environments. Table 3, Table 4, Table 5 and Table 6 report the remaining dry mass after exposure, whereas Figure 4 presents the same measurements converted into percentage mass loss to provide a graphical overview of the temporal trends. The plotted values represent the mean results, and the error bars represent the standard deviation of the triplicate measurements.

The rPET fibers showed mass losses ranging from 0 to 0.08% across all solutions and exposure periods. This result indicates very high mass retention rather than an absolute absence of mass change. In contrast, natural wool showed approximately 20% mass loss in bleach after 24 h and moderate mass losses in liquid soap and dilute HCl after longer exposure periods. Cotton remained stable in powder detergent, liquid soap, and bleach but showed approximately 10% mass loss in dilute HCl after 3 days. Apparel fabric showed approximately 10% mass loss in bleach and dilute HCl after 24 h, while a similar loss developed in liquid soap after 3 days.

These results show that rPET retained its dry mass under the tested conditions. However, the reference materials differed in composition, structure, and physical form. Their results therefore provide descriptive context and should not be interpreted as directly equivalent degradation kinetics.

### 3.3. Behavior of Apparel Fabric

Table 3 presents the remaining dry mass of the apparel fabric samples after each exposure period. The fabric maintained nearly its initial mass in powder detergent throughout the experiment. In liquid soap, the mass remained nearly unchanged for 24 h but decreased to approximately 9.01 g after 3 and 7 days. In bleach and dilute HCl, the mass decreased to approximately 9.01 g after 24 h and remained at a similar level during longer exposure. The low standard deviation values indicate good repeatability among the triplicate measurements.

The observed mass reduction may reflect the removal or degradation of one or more components of the polyester and cotton blend. However, the respective contributions of the two constituents cannot be identified from mass measurements alone.

### 3.4. Behavior of Cotton Batting

Table 4 presents the remaining dry mass of the cotton batting samples. The mass remained nearly unchanged in powder detergent, liquid soap, and bleach throughout the exposure period. In contrast, exposure to dilute HCl resulted in a final mass of approximately 9.01 g after 3 and 7 days, corresponding to a mass loss of approximately 10%.

This pattern is consistent with the known sensitivity of cellulose to strongly acidic environments, where cleavage of glycosidic bonds may occur. However, this mechanism was not directly examined using molecular or structural characterization methods in the present study.

### 3.5. Behavior of Natural Wool

Table 5 summarizes the remaining dry mass of the natural wool samples. Natural wool exhibited its largest mass change during bleach exposure. The mass decreased to 8.012 ± 0.055 g after 24 h, corresponding to approximately 20% mass loss, and remained at a similar level during longer exposure periods. Moderate mass losses of approximately 9% were observed in liquid soap after 3 days and in dilute HCl after 24 h.

The mass remained nearly unchanged in powder detergent under the tested conditions of 25 ± 1 °C, static immersion, and a maximum exposure time of 7 days. This result describes the present experimental measurements and should not be generalized to other detergent compositions, pH values, temperatures, or washing conditions.

The pronounced mass reduction in bleach is compatible with oxidative damage to wool keratin, while the response to dilute HCl may reflect acid-related degradation. Nevertheless, these mechanisms were not directly verified through chemical or structural analyses.

### 3.6. Behavior of Recycled PET Fibers

The mass stability of recycled PET fiber samples is reported in Table 6. The remaining mass ranged from 9.992 to 10.000 g, corresponding to mass retention between 99.92 and 100%. The low standard deviation values indicate good repeatability across the triplicate measurements.

The limited mass changes observed at 25 ± 1 °C may be related to restricted penetration of the aqueous media and limited access to the ester bonds of PET under the static exposure conditions. The slightly greater changes observed in bleach and dilute HCl after 7 days may indicate a limited time dependent response. However, the magnitude of these changes remained below 0.1%.

These results demonstrate high mass retention of the rotary-die-extruded rPET fibers under the tested conditions. They do not independently confirm the degree of crystallinity, molecular orientation, or absence of molecular chain cleavage because X ray diffraction, scanning electron microscopy, FTIR, DSC, and GPC analyses were not performed.

### 3.7. Impact of Chemical Exposure on Mechanical Integrity

Although the rPET fibers showed only negligible mass changes, mechanical testing was conducted to determine whether their tensile properties changed after exposure. As shown in Table 7, tensile strength decreased from 245.5 ± 5.2 MPa for the untreated fibers to 235.2 ± 5.5 MPa after 7 days in dilute HCl, corresponding to a strength retention of 95.8%. Exposure to bleach resulted in a strength retention of 97.2%.

Young’s modulus decreased from 2.85 ± 0.12 GPa to 2.70 ± 0.13 GPa after dilute HCl exposure. Elongation at break decreased from 28.4 ± 2.1% to 24.8 ± 1.9%. These results indicate that the tensile properties were largely retained, although small reductions were observed in the more aggressive solutions.

As demonstrated in Table 7, the mechanical integrity of rPET fibers remains remarkably high across all chemical environments. Strength retention exceeded 95% in all tested solutions. This finding suggests that no major loss of load bearing capacity occurred during the 7 day exposures. However, tensile property retention does not directly prove that the ester bonds were unaffected or that molecular chain cleavage was completely absent.

Regarding surface morphology, visual and optical microscopic examinations of the rPET fibers after 7 days of immersion revealed no perceptible signs of degradation, such as fibrillation, pitting, or surface cracking; the fibers maintained their original transparency and smooth appearance. These observations indicate that no major surface damage was visible at the resolution of the optical method. Because scanning electron microscopy was not performed, subtle changes at smaller length scales cannot be excluded.

The retention of tensile strength is consistent with the absence of major optically visible defects because severe surface damage would generally reduce tensile performance. Nevertheless, the combination of optical microscopy and tensile testing cannot provide direct evidence of molecular chain integrity, functional group preservation, or crystallinity.

Taken together, the mass retention and tensile results indicate that the rotary-die-extruded rPET fibers maintained most of their measured properties under the selected aqueous conditions. These findings describe the performance of the investigated rPET fibers and do not establish mechanical superiority over wool, cotton batting, or apparel fabric because equivalent tensile tests were not conducted for those materials.

To provide an integrated overview of the chemical resistance behavior, the results obtained for all textile materials in the different chemical environments were synthesized into a qualitative comparison, as summarized in Table 8. The categories in Table 8 are based only on the measured mass changes and do not represent equivalent degradation mechanisms among the different materials.

The findings indicate that the investigated rPET fibers retained their mass and most of their tensile properties during static exposure at room temperature. This behavior supports their potential use in textile applications requiring resistance to the tested aqueous environments. However, the experiments do not reproduce complete domestic or industrial washing conditions because agitation, repeated washing cycles, and exposure at 60 °C were not investigated.

The comparison with wool, cotton batting, and apparel fabric should remain descriptive because these materials differed in composition, geometry, and surface area to volume ratio. In addition, the pH and active chlorine concentration of the commercial solutions were not monitored throughout the exposure period. Possible changes in solution activity over time, particularly during bleach exposure, may therefore have influenced the results.

Overall, the present results demonstrate high mass retention and tensile property retention for the rotary-die-extruded rPET fibers under the investigated conditions. Further work should examine realistic detergent concentrations, exposure at 60 °C, repeated washing cycles, solution stability, virgin PET controls, and direct structural characterization to verify the mechanisms responsible for the observed behavior.

## 4. Conclusions

This study evaluated the chemical stability of recycled PET fibers produced by rotary die extrusion under exposure to powder detergent, liquid soap, bleach, and dilute hydrochloric acid for periods ranging from 2 h to 7 days. Natural wool, cotton batting, and apparel fabric were included as descriptive reference materials, while mechanical testing was conducted only for the rPET fibers. The findings demonstrate that:

1. Recycled PET fibers showed negligible mass loss and retained more than 95% of their initial tensile strength after 7 days of exposure. These findings indicate high mass retention and mechanical property retention under the tested conditions. However, they do not directly confirm the absence of molecular chain cleavage or changes in crystallinity.
2. Natural wool and cotton batting showed greater mass changes under specific exposure conditions. Wool exhibited its largest mass loss in bleach, while cotton showed measurable mass loss in dilute hydrochloric acid. Because these materials differed from rPET in composition and physical form, the comparisons should be interpreted only as descriptive observations.
3. Apparel fabric showed measurable mass loss in bleach and dilute hydrochloric acid after 24 h and in liquid soap after longer exposure. Its response reflects the behavior of the tested polyester and cotton blend and cannot be directly compared with the degradation kinetics of loose rPET fibers.

Overall, the results demonstrate that the investigated rPET fibers retained most of their measured mass and tensile properties under the selected aqueous exposure conditions. This behavior supports their potential use in textile applications requiring resistance to the tested chemical environments. However, the high detergent concentrations and static exposure at 25 °C do not directly reproduce practical laundering conditions. This study also did not include a virgin PET control, exposure at 60 °C, repeated washing cycles, agitation, continuous monitoring of solution pH or active chlorine, or equivalent mechanical testing of the reference materials.

Future studies should therefore evaluate realistic detergent concentrations, solution renewal, repeated washing at elevated temperatures, and direct comparison with virgin PET fibers. Additional characterization using FTIR spectroscopy, differential scanning calorimetry, X ray diffraction, scanning electron microscopy, and gel permeation chromatography would also help determine whether chemical exposure causes changes in functional groups, crystallinity, surface morphology, or molecular weight.

## Figures and Tables

**Table 1:** Typical physical and chemical properties of polyethylene terephthalate.

Property	Value
Formula	(C_10_H_8_O_4_)*_n_*
Glass Transition Temperature	70 to 85 °C
Melting Temperature	245 to 260 °C
Enthalpy of Fusion	140 J/g
Decomposition Temperature	425 to 445 °C
Young’s Modulus	2100 to 3100 MPa
Coefficient of Linear Thermal Expansion	80 to 100 × 10^−6^ · K^−1^
Specific Heat Capacity	1.04 to 1.17 J/(g·K)
Thermal Conductivity	0.24 W/(m·K)
Density	1.33 to 1.45 g/cm^3^
Morphology	Semi crystalline thermoplastic

**Table 2:** Specifications of the aqueous solutions used in the resistance tests.

Solution	Composition and Concentration
Powder Detergent (OMO)	Commercial powder detergent, 100 g/L in water
Liquid Soap (LINA)	Commercial liquid soap, 100 g/L in water
Bleach (BREF)	Commercial chlorine bleach, 13° active chlorine
Hydrochloric Acid (NASSAH)	Diluted to 50% *v*/*v* in water, with an estimated pH below 1

**Table 3:** Remaining dry mass of apparel fabric samples in grams as a function of exposure time and solution type, *n* = 3, mean ± SD.

Time/Solution	Powder Detergent	Liquid Soap	Bleach	Dilute Hydrochloric Acid
2 h	10.000 ± 0.002	10.000 ± 0.002	10.000 ± 0.003	10.000 ± 0.002
24 h	10.000 ± 0.003	9.998 ± 0.005	9.025 ± 0.042	9.018 ± 0.038
3 days	9.998 ± 0.004	9.012 ± 0.035	9.015 ± 0.045	9.010 ± 0.040
7 days	9.997 ± 0.005	9.010 ± 0.038	9.008 ± 0.048	9.005 ± 0.042

**Table 4:** Remaining dry mass of cotton batting samples in grams as a function of exposure time and solution type, *n* = 3, mean ± SD.

Time/Solution	Powder Detergent	Liquid Soap	Bleach	Dilute Hydrochloric Acid
2 h	10.000 ± 0.001	10.000 ± 0.002	10.000 ± 0.002	10.000 ± 0.002
24 h	10.000 ± 0.002	10.000 ± 0.003	10.000 ± 0.003	9.998 ± 0.005
3 days	9.998 ± 0.004	9.997 ± 0.005	10.000 ± 0.004	9.015 ± 0.032
7 days	9.997 ± 0.005	9.995 ± 0.006	9.998 ± 0.005	9.012 ± 0.035

**Table 5:** Remaining dry mass of natural wool samples in grams as a function of exposure time and solution type, *n* = 3, mean ± SD.

Time/Solution	Powder Detergent	Liquid Soap	Bleach	Dilute Hydrochloric Acid
2 h	10.000 ± 0.002	10.000 ± 0.002	9.985 ± 0.012	10.000 ± 0.003
24 h	10.000 ± 0.003	9.995 ± 0.006	8.012 ± 0.055	9.155 ± 0.045
3 days	9.998 ± 0.004	9.120 ± 0.040	8.008 ± 0.058	9.142 ± 0.042
7 days	9.997 ± 0.006	9.115 ± 0.042	8.005 ± 0.062	9.138 ± 0.045

**Table 6:** Remaining dry mass of recycled PET fiber samples in grams as a function of exposure time and solution type, *n* = 3, mean ± SD.

Time/Solution	Powder Detergent	Liquid Soap	Bleach	Dilute Hydrochloric Acid
2 h	10.000 ± 0.001	10.000 ± 0.001	10.000 ± 0.002	10.000 ± 0.002
24 h	10.000 ± 0.002	10.000 ± 0.002	9.998 ± 0.003	9.997 ± 0.004
3 days	10.000 ± 0.002	9.998 ± 0.003	9.997 ± 0.004	9.995 ± 0.005
7 days	9.998 ± 0.003	9.997 ± 0.004	9.995 ± 0.005	9.992 ± 0.006

**Table 7:** Mechanical properties and strength retention of rPET fibers after 7 days of chemical exposure.

Solution Type	Tensile Strength (MPa)	Young’s Modulus (GPa)	Elongation at Break (%)	Strength Retention (%)
Control (Untreated)	245.5 ± 5.2	2.85 ± 0.12	28.4 ± 2.1	100.0%
Powder Detergent	243.2 ± 4.8	2.82 ± 0.10	27.9 ± 1.8	99.1%
Liquid Soap	241.8 ± 5.1	2.80 ± 0.11	27.5 ± 2.0	98.5%
Bleach (13° chlorine)	238.7 ± 6.1	2.75 ± 0.15	25.2 ± 2.4	97.2%
Dilute HCl (pH < 1)	235.2 ± 5.5	2.70 ± 0.13	24.8 ± 1.9	95.8%

**Table 8:** Summary Comparison of Chemical Resistance.

Textile Material	Powder Detergent	Liquid Soap	Bleach	Dilute Hydrochloric Acid	Overall Mass Loss
Recycled PET Fibers	Negligible	Negligible	Negligible	Negligible	Negligible
Natural Wool	Negligible	Moderate	High	Moderate	High
Cotton Batting	Negligible	Negligible	Negligible	Moderate	Moderate
Apparel Fabrics	Negligible	Moderate	Moderate	Moderate	Moderate

## Data Availability

The data that support the findings of this study are available on request from the authors.
